# A Case of Paraneoplastic Guillain-Barré Syndrome Associated with Squamous Cell Carcinoma of the Lung

**DOI:** 10.7759/cureus.3202

**Published:** 2018-08-24

**Authors:** Danwei Wu, Anne Liu, Esther Baldinger, Alfred T. Frontera

**Affiliations:** 1 College of Medicine, University of Central Florida, Orlando, USA; 2 Neurology, Bay Pines VA, Bay Pines, USA

**Keywords:** guillain-barré syndrome, paraneoplastic, squamous cell cancer, lung cancer

## Abstract

We report a case of a 61-year-old man with a history of squamous cell carcinoma of the lung presenting with rapidly progressive symmetric ascending weakness with areflexia. The weakness was quickly followed by respiratory decompensation requiring intubation. Lumbar puncture yielded cerebrospinal fluid with elevated protein (177 mg/dL), normal glucose (61 mg/dL), normal red blood cell count (0 per/µl), and normal white blood cell count (0 per/µL). Emergent magnetic resonance imaging of cervical, thoracic, and lumbar spine did not show evidence of metastatic disease, fracture, subluxation, or other causes of cord compression. The patient was diagnosed with acute inflammatory polyneuropathy, also known as Guillain-Barré syndrome. Despite treatment with a five-day course of intravenous immunoglobulin and a subsequent five-day course of plasmapheresis, the patient did not recover respiratory function and died 48 days after diagnosis. To our knowledge, this is the first documented case of Guillain-Barré occurring concomitantly with squamous cell carcinoma of the lung.

## Introduction

Guillain-Barré syndrome (GBS) is an acute paralytic neuropathy characterized by symmetric, ascending weakness and areflexia. GBS is the most common cause of acute flaccid paralysis with a reported incidence of 0.89 to 1.89 cases per 100,000 persons per year. In typical cases, the onset occurs secondary to respiratory or gastrointestinal infection. The pathogenesis is thought to be due to antibodies generated during infection that also bind to epitopes on the peripheral nervous system [1].

Paraneoplastic syndromes refer to a broad array of illnesses that damage organs or tissues distal to the neoplasm. These syndromes are not directly caused by the malignancy but rather an off-target effect of the immune response generated against the malignancy. Currently, it is believed that most if not all paraneoplastic neurologic syndromes are immune-mediated. Paraneoplastic neurologic disorders can involve all portions of the nervous system. Although paraneoplastic syndromes are rare, affecting only 0.01% of patients with cancer, there are some common paraneoplastic syndromes that affect the nervous system including Lambert-Eaton syndrome, which affects 3% of patients with small-cell lung cancer, and myasthenia gravis which affects about 15% of patients with thymoma [2].

The presence of paraneoplastic GBS remains controversial but there are reports of GBS occurring in the presence of different types of cancers including lung cancer [3-10], non-lung squamous cell carcinoma [11], gastrointestinal cancers [12,13], bladder cancer [14], and lymphomas [15,16]. Here, we report a case of a 61-year-old man with untreated squamous cell carcinoma of the lung diagnosed with rapidly progressing GBS. This is the first case, to our knowledge, of GBS associated with squamous cell carcinoma of the lung.

## Case presentation

A 61-year-old man was admitted for a one-day history of progressive bilateral ascending lower-limb weakness and sensory deficits. The patient had a 40 pack-year smoking history. Three months prior to admittance, the patient was diagnosed with squamous cell lung cancer. The patient had no neurologic complaints at the initial diagnosis of cancer. At the time of diagnosis, computed tomography (CT) scan of the chest showed 9.0 cm left upper lobe mass with central necrosis. A positron emission tomography (PET) scan revealed two hypermetabolic perivascular lymph nodes as well as periportal and aortocaval lymph node consistent with malignancy. Biopsy and staging of the cancer revealed poorly differentiated stage IV (T4, N3, M1) squamous cell carcinoma.

At initial presentation, the patient was alert and oriented with new onset of weakness in his lower extremities requiring the use of a cane. The patient also complained of sensory deficits in his legs and fingertips beginning since the morning of his admission. On physical exam, the patient was alert and responsive. Cranial nerves were intact. Strength testing demonstrated 4/5 weakness in hip flexors and extensors bilaterally. The upper extremities showed 5/5 strength. Deep tendon reflexes could not be elicited. No fasciculations of muscles were observed. Sensory testing revealed decreased response to light touch below the knee bilaterally. The patient did not exhibit dysdiadochokinesia or dysmetria.

By the afternoon of the second hospital day, the weakness had worsened to involve the arms symmetrically. The patient was intubated to protect his airway. Strength in lower extremities was 1/5 bilaterally. The upper extremities were 2/5 bilaterally. The next day, all extremities were flaccid, the patient was respirator dependent with facial weakness. The patient was still able to respond to voice through blinking and eye movements.

Imaging

**Figure 1:**
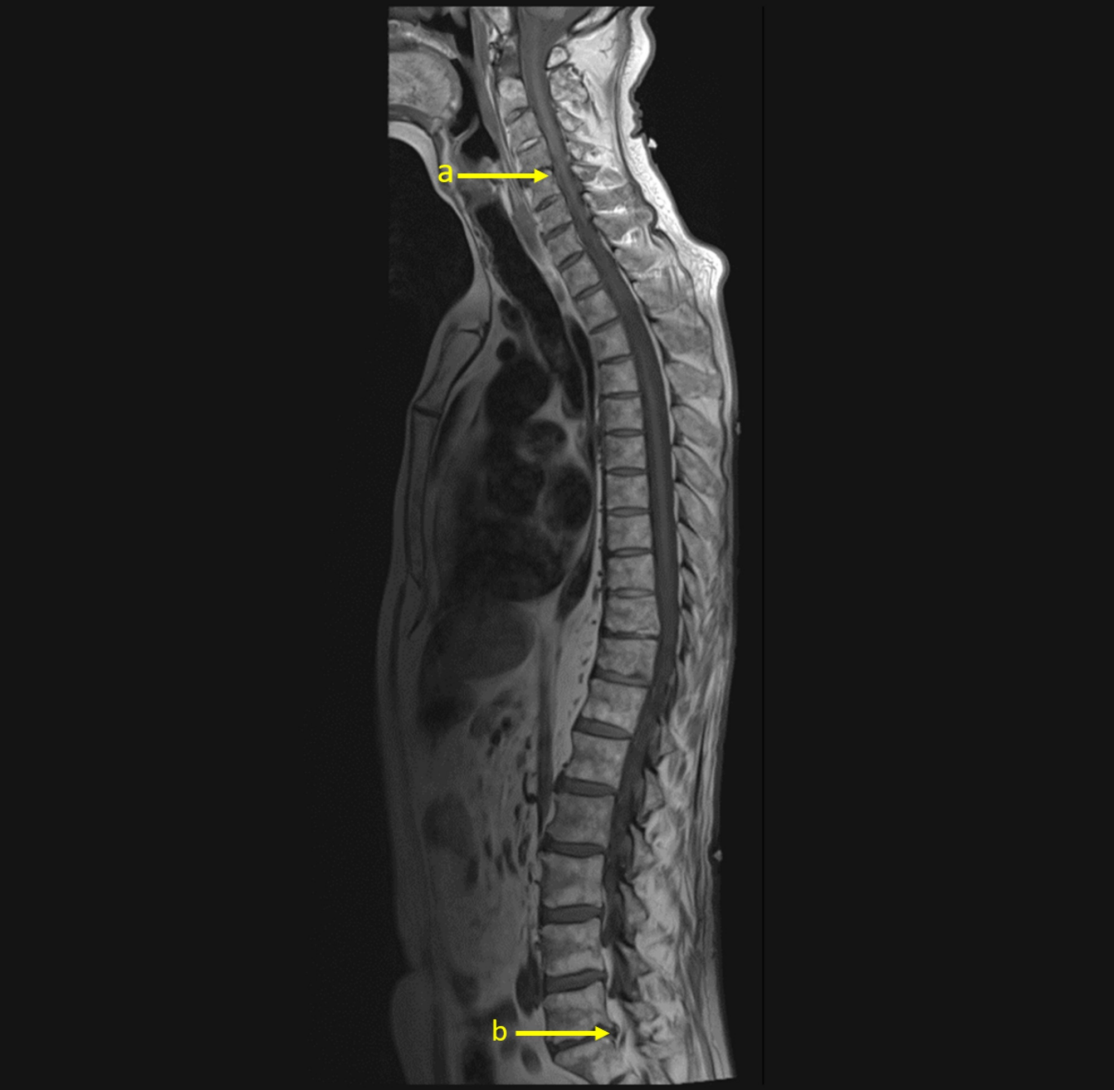
T1-weighted magnetic resonance imaging (MRI) without contrast of whole spine. T1-weighted sagittal MRI without contrast of complete spine demonstrating absence of fracture, subluxation, and abnormal cord signal in cervical, thoracic, and lumbar spine. a) Disc osteophyte complex present at C4-C5 contacting thecal sac with probable mild-moderate spinal canal stenosis. b) Moderate bilateral neural foraminal narrowing at L5-S1 present.

Emergent magnetic resonance imaging (MRI) of whole spine without contrast showed absence of fracture, subluxation, and abnormal cord signal. Imaging was negative for both metastatic disease and spinal cord compression (Figure 1). CT head scan without contrast was also negative for metastatic disease. Additional neuroimaging was unable to be obtained after intubation.

Investigation

On admission the patient had normal complete blood count. Hypokalemia was present (2.7 mEq/L). Ferritin was elevated (631.7 ng/ml). Renal, hepatic, and thyroid functions were normal. B12 was on the lower border of normal (191 pg/ml), however, the patient had normal methylmalonic acid (143 nmol/L).

Additional labs were ordered to rule out other potential causes of acute polyneuropathies such as HSV, Lyme disease, vasculitis, and sarcoidosis. Polymerase chain reaction of cerebrospinal fluid (CSF) was negative for HSV1, HSV2, and Lyme DNA. Additional lab tests within normal range included erythrocyte sedimentation rate, C-reactive protein, angiotensin converting enzyme, and anti-MAG antibodies. The patient was negative for anti-GM1 antibodies. Additional antibodies specific to other subtypes of GBS were not ordered.

Gram stain and bacterial culture of CSF fluid was negative. In addition, patient was afebrile throughout the hospitalization and did not have symptoms suggestive of bacterial meningitis.

A lumbar puncture yielded clear CSF with protein (177 mg/dL), glucose (61 mg/dL), red blood cell count (0 per/µL), and white blood cell count (0 per/µL). CSF demonstrated albuminocytologic dissociation typical of Guillain-Barre. Nerve conduction studies could not be conducted.

Treatment

A course of intravenous immunoglobulins at 2 gm/kg of body weight was started for five days beginning on day two of admission. In addition, intramuscular B12 supplementation was started due to borderline low B12 levels. The patient was monitored and did not show signs of improvement. After a period of 10 days of observation with no changes in respiratory and motor status, additional treatment was felt to be necessary. Literature suggested that subtypes of Guillain-Barre with axonal involvement may respond better to plasmapheresis [17-19]. Therefore, a trial with plasmapheresis was initiated due to the lack of response to the intravenous immunoglobin. The patient underwent five days of plasma exchange at 3 L/day for the first three days followed by plasma exchange every other day for two more days. Despite treatment, there were no neurologic improvements.

Outcome

The patient did not recover from his weakness and could not tolerate being weaned off of the ventilator. Hospice care was offered, however, he passed away in the intensive care unit 48 days after diagnosis after agreeing to terminate respiratory support. 

## Discussion

Diagnosis of GBS is made primarily with clinical evaluation. Required features for diagnosis include progressive weakness of the legs and arms along with areflexia/decreased reflexes in the affected limbs. Additional supporting features include progressive symptoms over days to weeks, symmetrical distribution, autonomic dysfunction, primarily motor involvement, elevated protein in CSF with a cell count ≤50/mm^3^, and electrodiagnostic abnormalities. In this case study, the patient presented with the typical features of GBS [1].

Two-thirds of cases of GBS are preceded by upper respiratory tract infection or diarrhea. Common infectious agents associated with GBS include Campylobacter jejuni, cytomegalovirus, Epstein-Barr virus, varicella-zoster virus, and Mycoplasma pneumoniae. GBS is thought to occur secondary to post-infectious immune response, in which antibodies generated by the body also recognize components of the peripheral nervous system termed molecular mimicry [1]. There is still controversy over whether GBS can occur as part of a broader spectrum of immune-mediated paraneoplastic neurologic disorders. Approximately 50% of patients diagnosed with paraneoplastic neurological syndromes are positive for onconeural antibodies, therefore, the absence of onconeural antibodies does not exclude the presence of a paraneoplastic involvement. However, there are documented cases involving all components of the nervous system, suggesting that onconeural antibodies can target different regions with varying propensity [2].

There are currently nine reported cases involving lung cancer [3-11]. Of the reported cases, seven involve small cell lung cancer [3,5-9,11] and two involve adenocarcinoma of the lung [4,10]. There is only one other documented case involving squamous cell, however the primary tumor originated from the mandible [20]. The patient in the reported case of GBS occurring concomitantly with squamous cell was diagnosed with cancer after the onset of ascending paralysis and similarly, did not respond to treatment with intravenous immunoglobulin. In our case, we document the onset of rapidly progressive GBS in the presence of squamous cell carcinoma of the lung refractory to both intravenous immunoglobulin and plasmapheresis. The aggressive progression of GBS associated with squamous cell carcinoma suggests that its pathogenesis may be different from classical forms of GBS, rendering immunosuppressive therapies such as intravenous immunoglobulin and plasmapheresis ineffective.

## Conclusions

In this report, we document a rare case of GBS with concomitant squamous cell carcinoma of the lung three months after diagnosis of malignancy. Similar to the previously reported case involving squamous cell of the mandible, neurologic symptoms were refractory to intravenous immunoglobulin. Additionally, the patient in this case was not responsive to plasmapheresis, suggesting that GBS secondary squamous cell is aggressive and difficult to treat. The presence of paraneoplastic GBS remains controversial. However, there are numerous case reports of GBS associated with malignancies in the absence of infection. Based on this evidence, investigation of refractory GBS should include workup of possible malignancy if other causes are negative. Further studies must be conducted to determine if other measures, such as decreasing tumor burden, could treat GBS associated with cancer in cases refractory to normal therapies.
